# Machine Learning Detects Pan-cancer Ras Pathway Activation in The Cancer Genome Atlas

**DOI:** 10.1016/j.celrep.2018.03.046

**Published:** 2018-04-03

**Authors:** Gregory P. Way, Francisco Sanchez-Vega, Konnor La, Joshua Armenia, Walid K. Chatila, Augustin Luna, Chris Sander, Andrew D. Cherniack, Marco Mina, Giovanni Ciriello, Nikolaus Schultz, The Cancer Genome Atlas Research Network, Yolanda Sanchez, Casey S. Greene

**Affiliations:** 1 Genomics and Computational Biology Graduate Group, Perelman School of Medicine, University of Pennsylvania, Philadelphia, PA 19104, USA; 2 Department of Systems Pharmacology and Translational Therapeutics, University of Pennsylvania, Philadelphia, PA 19104, USA; 3 Marie-Josée & Henry R. Kravis Center for Molecular Oncology, Memorial Sloan Kettering Cancer Center, New York, NY 10065, USA; 4 cBio Center, Department of Biostatistics and Computational Biology, Dana-Farber Cancer Institute, Boston, MA 02215, USA; 5 Department of Cell Biology, Harvard Medical School, Boston, MA 02115, USA; 6 The Eli and Edythe L. Broad Institute of Massachusetts Institute of Technology and Harvard University, Cambridge, MA 02142, USA; 7 Department of Medical Oncology, Dana-Farber Cancer Institute, Boston, MA 02215, USA; 8 Department of Computational Biology, University of Lausanne, Lausanne, Switzerland; 9 Department of Epidemiology and Biostatistics, Memorial Sloan Kettering Cancer Center, New York, NY 10065, USA; 10 Department of Molecular Systems Biology, Norris Cotton Cancer Center, Geisel School of Medicine at Dartmouth, Hanover, NH 03755, USA

## Abstract

Precision oncology uses genomic evidence to match patients with treatment but often fails to identify all patients who may respond. The transcriptome of these “hidden responders” may reveal responsive molecular states. We describe and evaluate a machine-learning approach to classify aberrant pathway activity in tumors, which may aid in hidden responder identification. The algorithm integrates RNA-seq, copy number, and mutations from 33 different cancer types across The Cancer Genome Atlas (TCGA) PanCanAtlas project to predict aberrant molecular states in tumors. Applied to the Ras pathway, the method detects Ras activation across cancer types and identifies phenocopying variants. The model, trained on human tumors, can predict response to MEK inhibitors in wild-type Ras cell lines. We also present data that suggest that multiple hits in the Ras pathway confer increased Ras activity. The transcriptome is underused in precision oncology and, combined with machine learning, can aid in the identification of hidden responders.

## In Brief

Way et al. develop a machine-learning approach using PanCanAtlas data to detect Ras activation in cancer. Integrating mutation, copy number, and expression data, the authors show that their method detects Ras-activating variants in tumors and sensitivity to MEK inhibitors in cell lines.

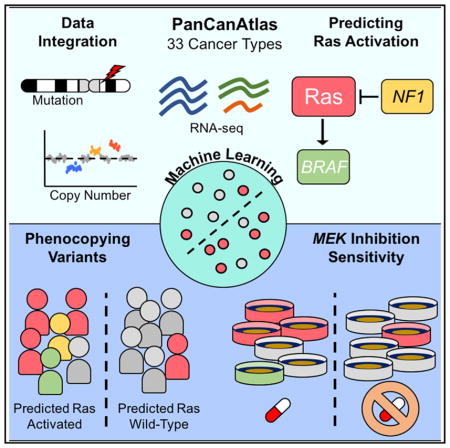

## INTRODUCTION

Precision oncology matches cancer patients to specific therapies based on genomic evidence, but it has benefited only a relatively low proportion of cancer patients to date (Prasad et al., 2016). While clinically promising, precision oncology lacks complete and accurate matching strategies and fails to identify many patients that could be matched using alternative approaches (Kumar-Sinha and Chinnaiyan, 2018). Cataloging transcriptome measurements across thousands of tumors enables a systems-biology perspective into the downstream consequences of molecular perturbation. Detecting these perturbations using transcriptomic states can improve precision oncology efforts toward more accurate and complete pairing of patients to effective treatments (Cieślik and Chinnaiyan, 2018).

In the largest uniformly processed cancer dataset to date, The Cancer Genome Atlas (TCGA) PanCancerAtlas has released multi-platform genomic measurements across thousands of tumors from 33 different cancer types (Weinstein et al., 2013). With this scale of data, researchers can build and evaluate statistical models that stratify tumors based on aberrant gene and pathway function. Previously, strategies have been explored using expression signatures to stratify patients (Bild et al., 2006). Some strategies have used data from individual cancer types. For example, gene expression signatures in colon adenocarcinoma (COAD) and glioblastoma (GBM) stratified tumors with aberrant *KRAS* and *NF1* function, respectively (Guinney et al., 2014; Way et al., 2017). Furthermore, data integration approaches incorporating pathway connectivity, including PARADIGM, are used to characterize pathway activity and infer gain- or loss-of-function events (Vaske et al., 2010; Ng et al., 2012; Sokolov et al., 2016). An unsupervised approach decomposing gene expression states in cell lines to map pathway activity has been proposed (Kim et al., 2017). Here, we introduce an elastic net penalized logistic regression classifier to learn signatures of gene or pathway alterations from gene expression assays of tumor biopsies across cancer types. We applied our method across cancer types to learn an independent, pan-cancer signature of pathway aberration. Our method can be used to identify phenocopying variants and requires only gene expression data for inference on new data. We apply our method to detect Ras pathway activation pan-cancer.

The Ras pathway is frequently altered in many different cancer types (De Luca et al., 2012). When the pathway is activated, often by gain-of-function *KRAS*, *NRAS*, or *HRAS* mutations or through *NF1* loss-of-function events, cells increase their translational output, and unchecked cellular proliferation occurs (McCormick, 1989; Xu et al., 1990). Certain cancer types, such as pancreatic adenocarcinoma (PAAD), skin cutaneous melanoma (SKCM), thyroid carcinoma (THCA), lung adenocarcinoma (LUAD), and COAD are known to be largely driven by mutations in Ras pathway genes (Goretzki et al., 1992; Omholt et al., 2003; Pao et al., 2005; di Magliano and Logsdon, 2013). Additionally, mutations in the Ras pathway have been observed to be early events driving tumorigenesis and have also been associated with poor survival and treatment resistance (Garcia-Rostan et al., 2003; Vauthey et al., 2013; Dinu et al., 2014; Hsu et al., 2016). Because the Ras pathway is ubiquitously misregulated, developing specific therapeutic targets is one of the National Cancer Institute’s key initiatives. However, Ras is also notoriously difficult to therapeutically target, and accurate detection of its malfunction is paramount (Stephen et al., 2014).

The most direct method of assessing Ras activation is by targeted sequencing of Ras. However, these methods would fail to detect unknown variants in other genes that phenocopy Ras-activating mutations. Detecting such tumors may enable more patients to be targeted therapeutically. In the present study, we describe our machine-learning approach that integrates bulk RNA sequencing (RNA-seq), copy number, and mutation data from the PanCanAtlas. We apply the method to Ras genes and demonstrate that our method can detect Ras activation pan-cancer. The classifier also identifies NF1 phenocopying events in TCGA and prioritizes Ras wild-type cell lines that respond to MEK inhibitors. Manually curated oncogenic variants in Ras pathway genes were assigned higher classification scores than variants with unknown significance. Our method can be applied to other cancer-associated genes and pathways as well. For example, the DNA Damage Repair PanCanAtlas analysis working group (AWG) applied this approach to detecting *TP53* inactivation (Knijnenburg et al., 2018).

## RESULTS

### Machine-Learning Models to Predict Pathway Activity

**Figure 1:**
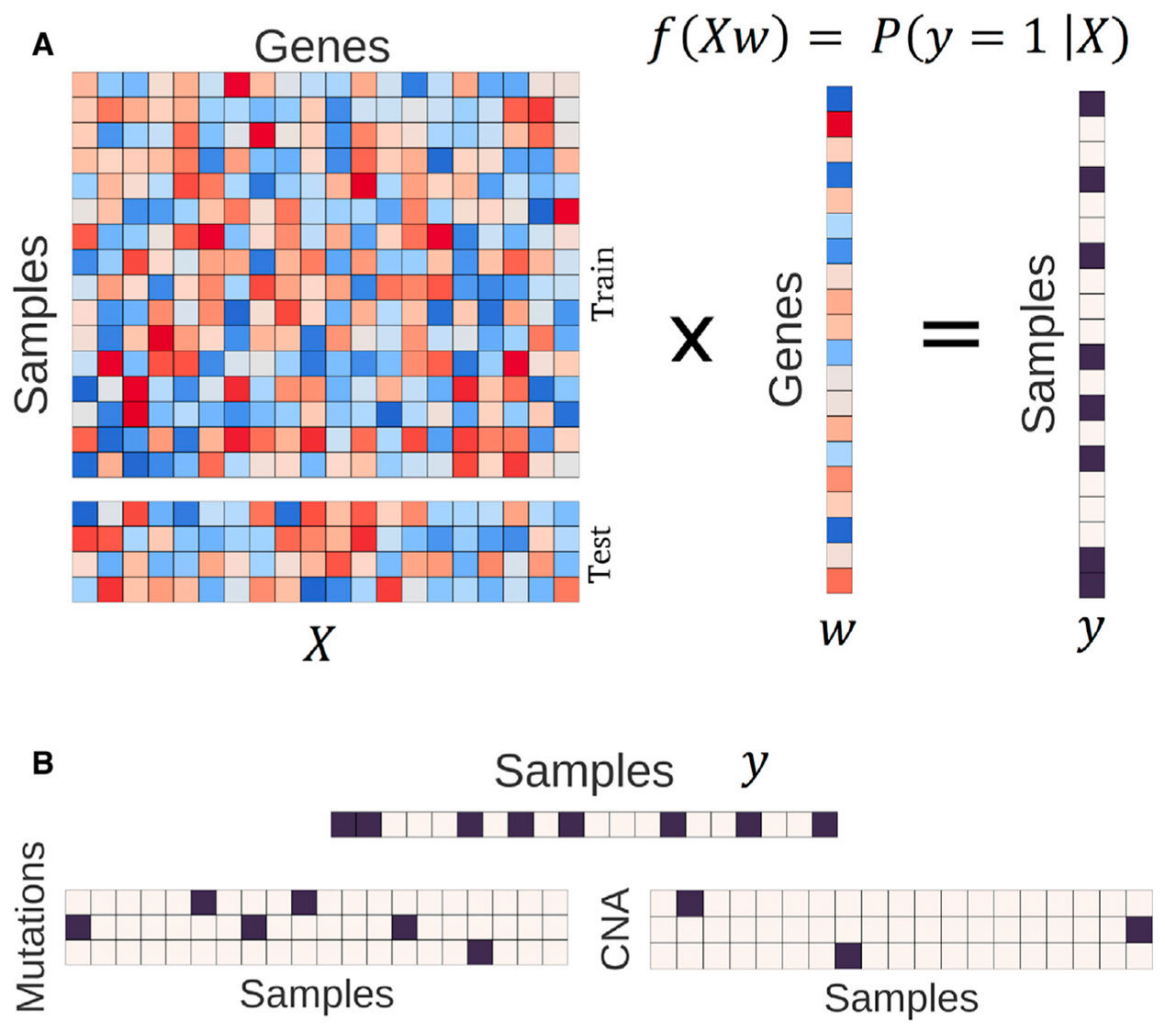
Framing the Algorithm and Integration Tasks (A) RNA-seq data (*X*) is multiplied by a vector of gene weights (*w*) where the optimization task is to find the optimal *w* to correctly classify the pathway status matrix (*y*). We train the model with the train partition and evaluate performance on a held-out test set. (B) The status matrix, *y*, is constructed by integrating mutations and copy number alterations (CNA). We consider activating or loss-of-function mutations and high copy number gain and deep copy number loss for oncogenes and tumor-suppressor genes, respectively. Black squares indicate aberrant events. For the Ras classifier, we used non-silent somatic mutations and high copy gains in the oncogenes *KRAS*, *NRAS*, and *HRAS*.

We developed a machine-learning approach to detect aberrant pathway activity in tumors. The method integrated RNA-seq, copy number, and mutation data. The models were trained using tumors from TCGA PanCanAtlas, with a complete set of these measurements, which included 9,075 tumors across 33 different cancer types. The method is based on a logistic regression classifier framework regularized with an elastic net penalty. We used RNA-seq as a measurement describing the expression state of a tumor and trained the classifier to detect downstream gene expression patterns consistent with aberrant pathway activity (Figure 1A). The algorithm learned a combination of gene importance scores, or weights (w), that together learn to best separate aberrant from wild-type expression patterns. As input during training, tumors with any non-silent somatic variants in target genes were included in the positive set (Figure 1B). We also included copy number gains for oncogenes and deep copy number loss for tumor suppressor genes (Figure 1B). For complete details about the model and training approach, refer to the STAR Methods. In principle, this approach could be applied to predict other gene or pathway events. Here, we applied the method to classifying Ras activity.

### Detecting Ras Activation Pan-cancer

We trained a classifier to detect aberrant Ras activity in tumors, using knowledge of *KRAS*, *HRAS*, and *NRAS* mutations and copy number gains (see Figure 1). These 3 core Ras genes differed greatly in variant prevalence across cancer types. In the PanCanAtlas, *KRAS* mutations were widespread in PAAD (72%), COAD (45%), rectum adenocarcinoma (READ, 42%), and LUAD (31%), while *NRAS* mutations were common in SKCM (31%) (Figure S1A). We performed a differential expression analysis of PanCanAtlas tumors, controlled for cancer type, comparing wild-type against aberrant Ras tumors (Figure S1B; Data S1).

**Figure 2:**
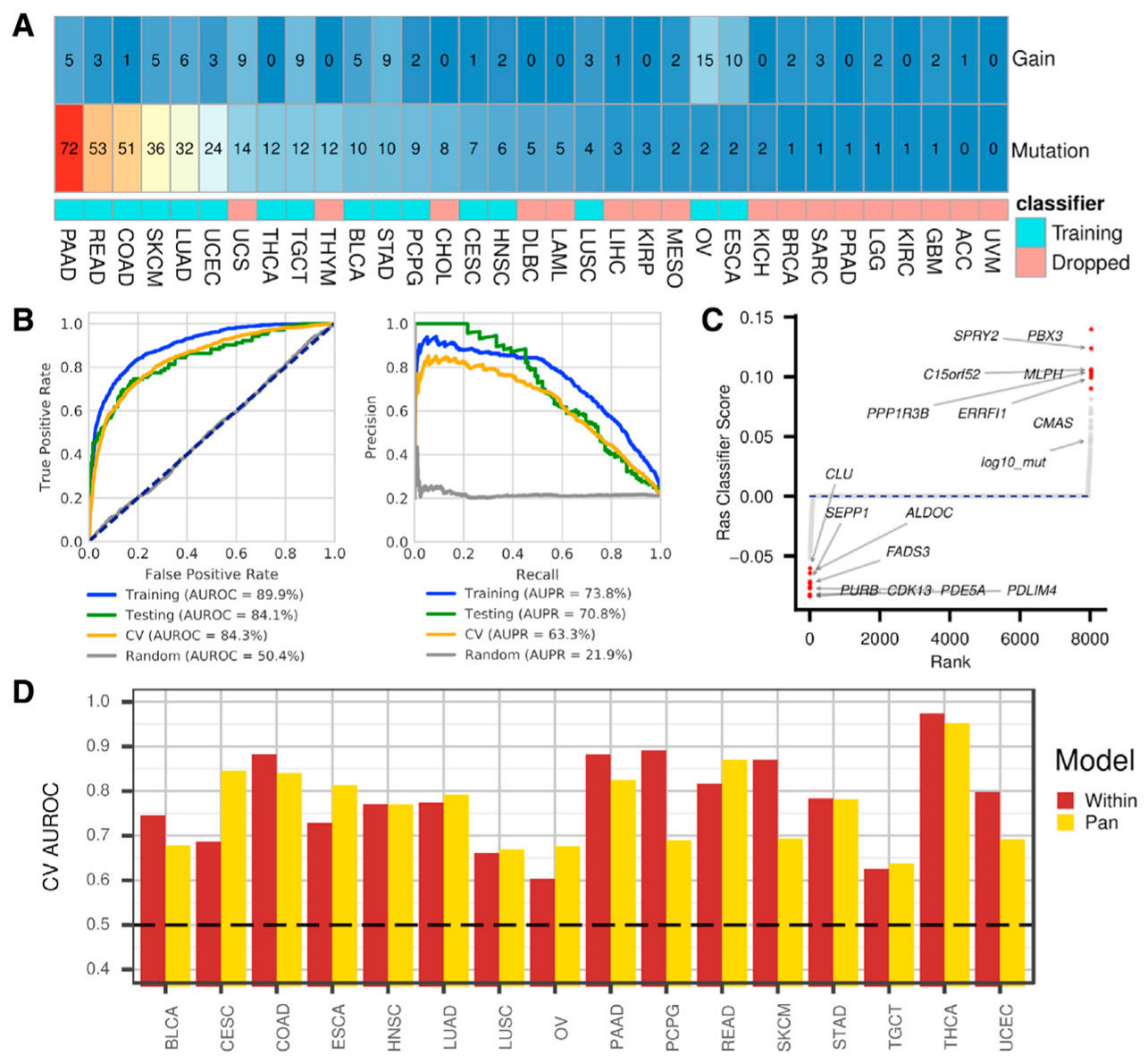
Evaluating Machine-Learning Classification of Ras Activation (A) Cancer-type-specific percentages of Ras aberration by copy number gain and deleterious mutation in *KRAS*, *HRAS*, or *NRAS*. The colored squares indicate whether the cancer type was included in model training. (B) Predicting Ras pathway activation metrics. The gray lines represent classifier predictions on a randomly shuffled gene expression matrix. Left: receiver operating characteristic (ROC) curve and area under the ROC (AUROC) curve given for training, testing, and cross-validation (CV) sets. The dotted navy line represents a hypothetical random classifier. Right: precision recall (PR) curve and corresponding area under the PR (AUPR) curve for each evaluation set. (C) Sparse classifier coefficients indicate which genes impact classifier performance. log10_mut represents tumor-specific non-silent mutation rate. (D) Cancer-type-specific performance for the pan-cancer model compared to separate models trained on each cancer type independently. See also Figures S2 and S3.

In the classifier, to enforce a more balanced class representation and to reduce performance metric inflation (Davis and Goadrich, 2006), we used samples from 16 of 33 cancer types for training (Figure 2A). We also used the top 8,000 most variably expressed genes by median absolute deviation (MAD) (see STAR Methods for details). We then randomly held out 10% of the samples (n = 476) to create a test set. The test set was selected to have the same proportion of cancer types and Ras statuses as the training set. The training set consisted of the remaining 90% (n = 4,283), which included 3,374 Ras wild-type tumors and 909 tumors with non-silent somatic Ras variants. Within the training set, we performed 5-fold cross-validation (CV). We report training (“training”), cross-validation (“CV”), and held-out test set (“testing”) performance using these cancer types. We also evaluated the final classifier on cancer types that were initially filtered from training.

Overall, the classifier showed high performance, with an area under the receiver operating characteristic (AUROC) curve above 84% and an area under the precision recall (AUPR) curve above 63% in the cross-validation and testing sets (Figure 2B). For the samples initially filtered from training, we also observed reasonable performance, with an AUROC curve of 75.2% and an AUPR curve of 24.7%. Therefore, the classifier detected Ras activation signal in tissues it was not exposed to during training. Applying the final classifier to all 9,075 samples, we observed an 86.7% AUROC curve and a 61.2% AUPR curve. We provide Ras prediction scores for each PanCancerAtlas sample in Data S2.

The Ras classifier consisted of automatically learned gene weights, or importance scores. Training with an elastic net penalty resulted in a sparse classifier, with only 185 genes contributing to classification. Genes and covariates with weights above zero can be interpreted as being upregulated in tumors with activated Ras, while negative-weight genes are characteristic of tumors with wild-type Ras (Figure 2C). The full classifier gene weights are provided in Data S3. However, caution must be exercised in interpreting these coefficients, as our elastic net regularization approach induces sparsity, which means that the solution represents a subset of genes associated with—and, therefore, useful for identifying—Ras activation. A differential expression analysis of Ras aberrant to wild-type tumors would reveal these downstream genes (Data S1).

Nevertheless, many of the classifier-implicated genes are known modulators of the Ras/MAPK (mitogen-activated protein kinase) pathway. For instance, high expression of *ERRFI1* contributed to predicting tumors with activated Ras. *ERRFI1* is a tumor suppressor of various receptors in the Ras pathway (Masoumi-Moghaddam et al., 2014). The top positive gene, *PBX3*, is a transcription factor previously implicated in certain astrocytomas (Ho et al., 2013b). The second top positive gene, *SPRY2*, inhibits FGFR signaling and interacts with *ERBB1*. The negatively associated genes are indicative of expression profiles of wild-type Ras tumors. For example, *CDK13* was the most predictive gene and is involved in regulating transcription, which potentially indicates an alternative mechanism driving transcriptional disruption in wild-type Ras tumors. We also compared pan-cancer classification with classifiers trained independently within each cancer type. Both the cancer-type-specific and pan-cancer classifiers had variable performance across cancer types, with the pan-cancer model outperforming the models optimized within cancer types approximately half of the time (Figure 2D).

### Ras Classifier Benchmarking Analyses

We performed several analyses to evaluate the robustness of the Ras classifier. A null model trained on a randomly shuffled gene expression matrix performed with about 50% AUROC and 20% AUPR in holdout test and cross-validation sets, which indicates strong performance of the model over this baseline (Figures S2A and S2B). We also assessed performance of the classifier for detecting Ras mutations and Ras copy number gains separately. Performance was similar, with the mutations-only model performing better than the combined model and the copy-number-only model performing worst (Figure S2C). Our model was robust to dropping *KRAS*, *NRAS*, and *HRAS* and 11 other Rasopathy genes from the gene expression matrix (Figure S2D). Lastly, performance was not impacted by covariate information (Figure S2E).

We also explored gene coefficient relationships across models. The high-weight-positive genes in the copy-only model included *C12orf11* (*ASUN*), *MRPS35*, *ERGIC2*, and *CMAS*, all of which are located on chromosome 12p near *KRAS*, which may indicate artifacts of common copy-gain events and be a result of low sample size in the positive-copy-only set (Figure S2F). Gene coefficients were similar across models when dropping different Ras pathway genes (Figure S2G). Lastly, we compared our machine-learning approach to a differential expression analysis of Ras mutant versus wild-type tumors controlled by cancer type. The differential expression scores aligned closely with the learned Ras classifier coefficients but identified many more genes than the sparse classifier (Figure S2H) (Data S1). In summary, the Ras classifier differed depending on data-type inclusion but was robust to input genes in the expression matrix, did not rely on covariate data, and included similar but fewer genes than a differential expression analysis.

### Detecting Ras Activation in Cell Lines

**Figure 3:**
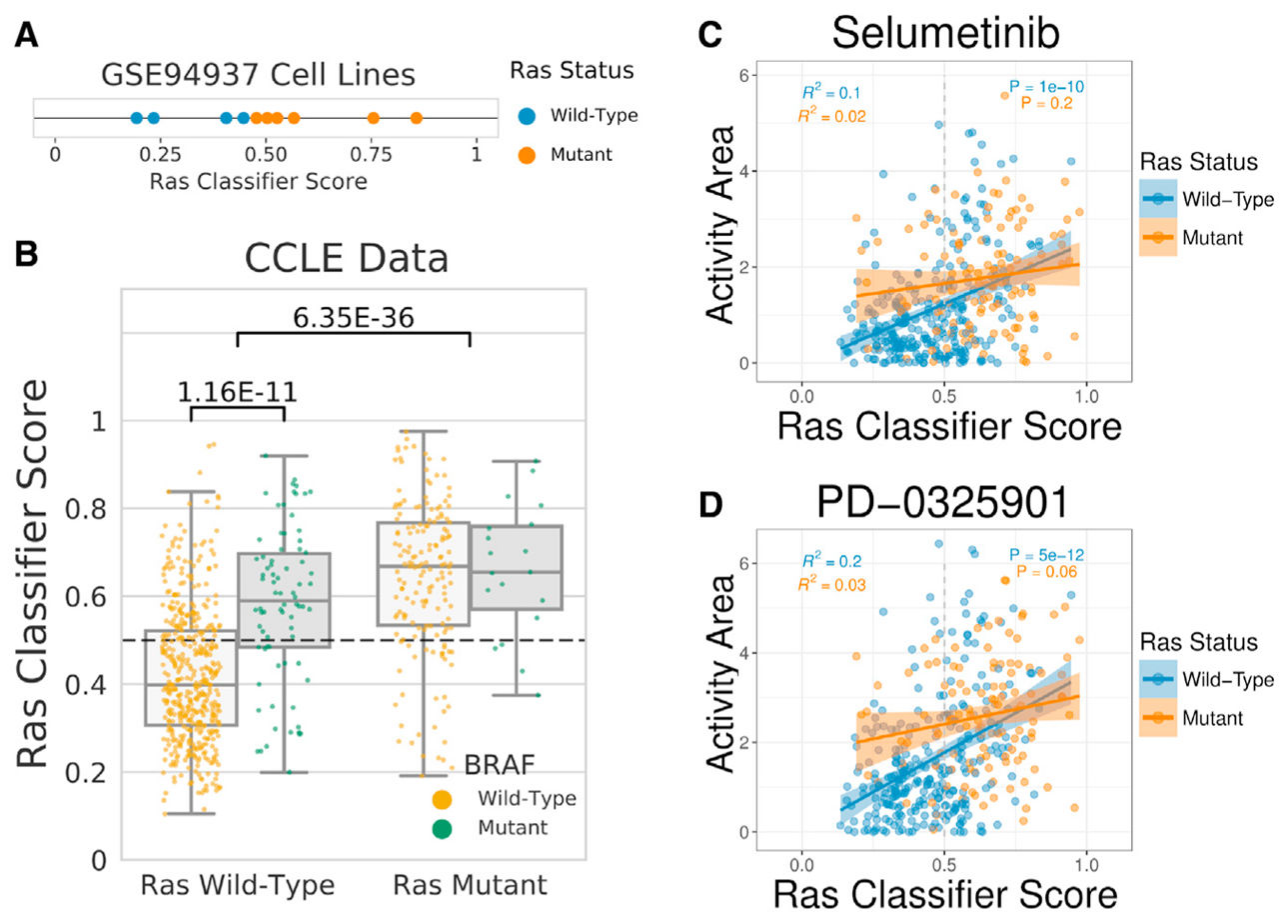
Cell-Line Predictions of Ras Activity (A) Ras classifier trained on PanCanAtlas tumors applied to a dataset of small airway epithelial cells (GEO: GSE94937). The mutant cells included a stably expressed *KRAS* G12V mutation. (B) Ras classifier trained on PanCanAtlas tumors applied to 737 cell lines from The Cancer Cell Line Encyclopedia (CCLE). Cell lines with *KRAS*, *HRAS*, or *NRAS* mutations are indicated in the right boxes, and wild-type tumors are indicated in the left boxes. Scores for cell lines with *BRAF* mutations (green) and wild-type *BRAF* (gold) are also shown. (C and D) Drug activity area for (C) selumetinib (AZD6244) and (D) PD-0325901 compared against Ras classifier scores for 388 CCLE cell lines with both gene expression and pharmacologic profiling data. Cell lines with mutant (orange) or wild-type (blue) *KRAS, HRAS,* and *NRAS* are indicated. The best fit lines, SE estimates, correlation coefficients, and p values are shown separately for cell lines with mutant or wild-type Ras.

We sought to determine whether predictions from the Ras classifier trained with TCGA tumors generalized to cell lines. We applied the classifier to two cell-line datasets. First, we applied the classifier to 10 small-airway epithelial cell RNA-seq profiles (GEO: GSE94937) (Kim et al., 2017). The set consisted of 4 wild-type profiles and 6 *KRAS* G12V-expressing mutant profiles. Our classifier correctly classified 9 out of 10 profiles and ranked all mutant profiles higher than all wild-type profiles (p = 1.16e–2) (Figure 3A). Though the PanCanAtlas data do not include gene-edited tumors that would allow us to directly evaluate Ras oncogenicity, the cell lines from this independent test set are induced to stably express a bona fide oncogenic *KRAS* variant.

Next, we applied our Ras classifier to RNA-seq profiles from 737 different cell lines from the Cancer Cell Line Encyclopedia (CCLE) with matched expression and mutation data (Barretina et al., 2012) (Figure 3B). The Ras classifier assigned significantly higher scores to Ras mutated (*KRAS*, *HRAS*, or *NRAS*) from Ras wild-type cell lines (p = 6.35e–36). Of the 393 cell lines predicted to be wild-type, 357 were labeled wild-type (negative predictive value = 90.8%). However, only 153 of 344 cell lines that were predicted to be Ras mutated were labeled Ras mutant (precision = 44.5%). In total, 510 of 737 (69.2%) cell lines were predicted correctly. In this case, the low precision could indicate either that the classifier failed to generalize or that the classifier successfully identified phenocopying events, which were negatives from the point of view of evaluations but also what we aimed to capture.

We sought to differentiate between these two possibilities by using independent information that was not provided to the classifier. First, we examined mutation status for *BRAF*, a well-characterized oncogene downstream of Ras genes (Davies et al., 2002). *BRAF* mutations that phenocopy Ras would be counted as negatives and, if they were highly ranked, would reduce the observed precision. Indeed, the classifier assigned significantly higher scores to *BRAF* mutant cell lines, compared to *BRAF* wild-type cell lines (p = 1.16e–11) (Figure 3B). Of all 191 false-positives, 56 had *BRAF* mutations (29.3%). The remaining false-positives indicated either tumors incorrectly assigned or tumors that harbored other phenocopying variants. Next, we tested CCLE pharmacological response data to determine whether Ras classifier scores were predictive of sensitivity to MEK inhibitors. We observed a strong correlation of the Ras classifier scores with sensitivity to two MEK inhibitors, selumetinib (AZD6244) and PD-0325901 (Figures 3C and 3D). The correlation was primarily driven by cell lines that were wild-type for Ras genes, implicating several drug-sensitive cell lines that may have otherwise been missed by direct sequencing of Ras genes. Taken together, the evaluation of additional mutations and the drug response data for Ras wild-type cell lines strongly suggested that the low precision in this case was related to the identification of phenocopying events.

Lastly, the classifier scored 34 cell lines harboring Ras mutations as Ras wild-type. We observed that 22 of these 34 false-negatives harbored variants annotated in the COSMIC database (64%) (Forbes et al., 2017). Conversely, 144 of 152 true-positives harbored COSMIC variants (95%), which is significantly higher than the proportion in false-negatives, χ^2^ = 26.1, degree of freedom 1, p = 3.2e–7. Therefore, our classifier detected signal at variant level resolution. We provide mean classifier scores for all nucleotide (Data S4) and amino-acid (Data S5) Ras variants observed in the CCLE.

### Other Ras Pathway Variants Phenocopy Ras Activation

**Figure 4:**
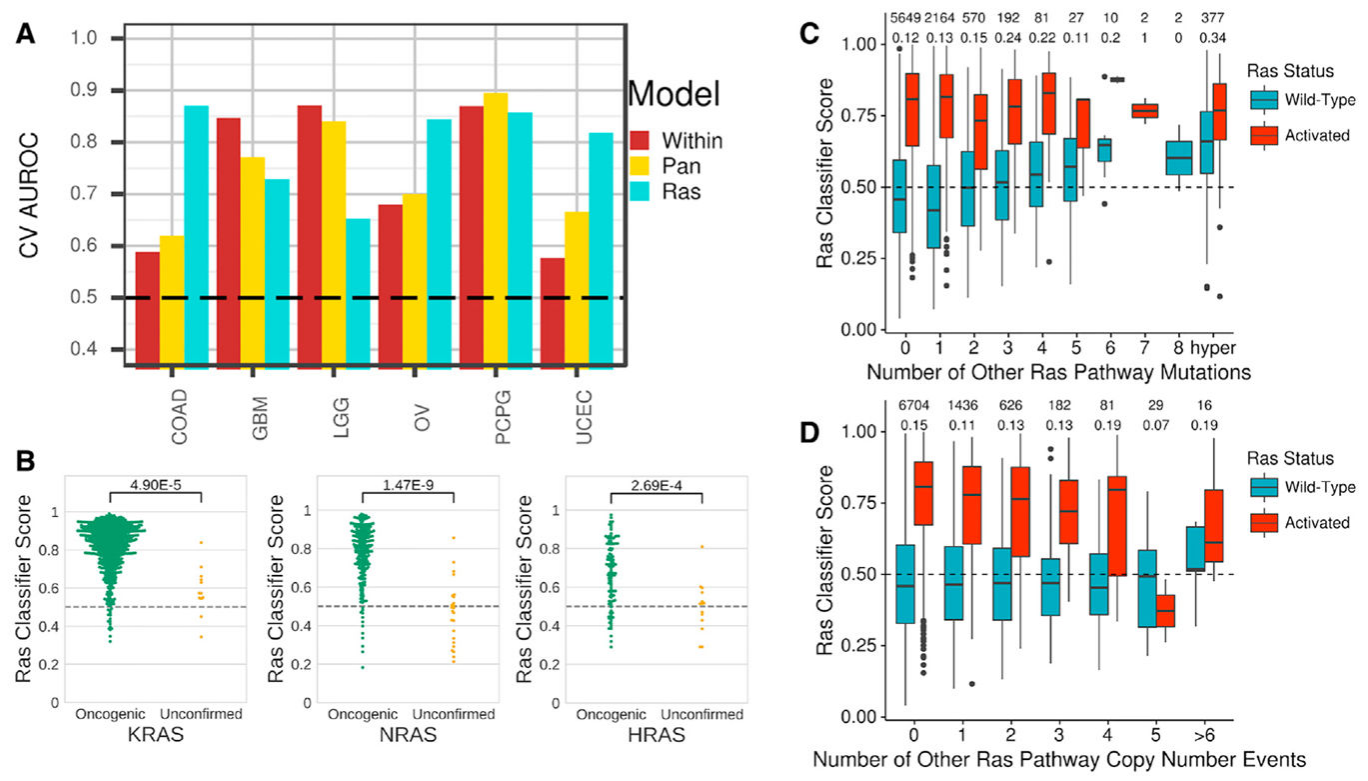
Ras Activation across Ras Variants and Alternative Ras Pathway Members (A) Cross-validation area under the receiver operating characteristic curve for predicting *NF1* inactivation. Within and pan-cancer models are classifiers trained to detect *NF1* inactivation. The Ras model is the classifier trained in Figure 2. The pan-cancer NF1 classifier is shown in Figure S3. (B) Ras classifier scores for samples with oncogenic or unconfirmed variants in *KRAS*, *HRAS*, and *NRAS*. Variant oncogenicity designations are based on curation (see STAR Methods). (C and D) Ras classifier scores stratified by Ras activity (*KRAS*, *NRAS*, *HRAS*) status and number of (C) aberrant mutations or (D) copy number alterations in other Ras pathway members. The two rows of numbers above each graph indicate number of samples in each group (top) and percentage of samples assigned to active Ras (bottom). See also Figure S3.

The Ras classifier was able to detect *NF1*-loss events particularly well in CNS tumors (GBM, low-grade glioma [LGG], and pheochromocytoma and paraganglioma [PCPG]). Performance was comparable to that of NF1 classifiers built using cancer-type-specific and pan-cancer models (Figure 4A). These tumors were not included in training the Ras classifier. Detection of *NF1*-inactivating events was also improved in COAD, OV, and uterine corpus endometrial carcinoma (UCEC), as compared to *NF1*-specific classifiers (Figure 4A). The Ras classifier’s performance predicting *NF1* loss of function was comparable to that of distinct pan-cancer models trained specifically to detect *NF1* loss-of-function events (Figure S3).

We applied the Ras classifier to curated variants in 38 core Ras pathway genes, which consisted of 34 oncogenes and 4 tumor-suppressor genes (Chakravarty et al., 2017; Sanchez-Vega et al., 2018). We provide Ras classifier scores for all Ras pathway mutations detected in PanCanAtlas tumors (Data S4 and Data S5). We observed an enrichment of high scores in tumors with oncogenic variants in *KRAS*, *NRAS*, and *HRAS* (Figure 4B). Scores for oncogenic *BRAF* variants were also enriched (Figure S4A). However, we noted that *BRAF* V600E mutations in THCA were overwhelmingly predicted to be Ras wild-type (Figure S4B). We trained a classifier for which we removed both of the *BRAF*-dominated cancer types (THCA and SKCM) (Figure S4C). In this model, we observed that THCA *BRAF* V600E mutations were predicted to have Ras activation, which aligns with previous understanding of *BRAF* function and our cell-line analysis (Figure S4D).

Lastly, in wild-type samples for *KRAS*, *NRAS*, and *HRAS* (Figure 4C, blue bars), we observed that Ras classifier scores increased after subsequent mutations in other pathway genes. In samples with a *KRAS*, *NRAS*, or *HRAS* mutation (Figure 4C, red bars), classifier scores did not increase after additional mutations to other genes in the pathway. However, more copy number events in other Ras pathway genes led to lower Ras classifier scores in Ras mutated samples (Figure 4D). These results potentially suggest that multiple hits in Ras pathway genes outside of Ras genes themselves may confer an increased Ras activation phenotype.

## DISCUSSION

We described a machine-learning method to detect malfunctioning genes and pathways in cancer and applied our method to detecting Ras activation. The method has variable performance across cancer types but is generally sensitive and specific overall, is generalizable to cell-line data, largely aligns with curated variant oncogenicity, and identifies phenocopying events leading to activated Ras. The approach can be applied generally to other genes and pathways.

The cell-line evaluation included accurately detecting isogenic lines transfected to express activating *KRAS* mutations and identifying CCLE cell lines with known Ras and *BRAF* mutations. We also demonstrated that CCLE Ras classifier scores were correlated with the drug activity of two MEK inhibitors (selumetinib and PD-0325901). In clinical trials, selumetinib did not increase overall survival in *KRAS* mutant advanced non-small-cell lung cancer (NSCLC) patients (Jänne et al., 2013, 2016). PD-0325901 also failed to meet efficacy endpoints in *KRAS* mutant NSCLC patients (Haura et al., 2010). Selumetinib and PD-0325901 have also been tested across many different cancer types, including ovarian, thyroid, skin, hepatocellular, breast, and colon cancers (Boasberg et al., 2011; Farley et al., 2013; Ho et al., 2013a; Jänne et al., 2016; O’Neil et al., 2011). Selumetinib has shown promising results in treating children with *NF1* mutant plexiform neurofibromas (Dombi et al., 2016), while PD-0325901 has shown efficacy in treating *NF1* mutant neurofibromas in mouse- and human-derived malignant peripheral nerve sheath xenografts (Jessen et al., 2013). Furthermore, the classifier automatically learns similar gene coefficients of an 18-gene panel previously curated using a targeted differential expression analysis to predict selumetinib sensitivity (Dry et al., 2010). Overall, our results suggest a useful biomarker application to potentially reveal hidden responders that may have otherwise been missed by sequencing.

Our approach to detecting Ras activation is supervised and, as with any supervised approach, is penalized by inaccurate labels. We encountered this limitation when detecting *BRAF* mutations in THCA. *BRAF* mutations are known to activate ERK and should not be classified as wild-type Ras (Oikonomou et al., 2014). Our results suggest that, in situations with predicted confounding mutations, it may be best to withhold a cancer type entirely during training. Withholding such data, as opposed to re-building a new classifier post hoc that uses *BRAF* V600E mutations as positive examples, may help to prevent a process of classifier creep, in which the classifier is continually expanded to improve metrics. Additionally, it is unclear how to best adjust for hypermutated phenotypes, as these tumors are more likely to have Ras mutations by chance. Unsupervised or semi-supervised methods to automatically retrieve gene expression signatures may overcome labeling issues and may sidestep some of the difficulties in modeling hypermutated tumors by first separating sources of variation.

While mutual exclusivity analyses across pathways drives hypotheses and reveals etiological insights (Babur et al., 2015; Mina et al., 2017), our findings suggest that, when multiple mutations occur in Ras pathway genes, tumors exhibit a transcriptional profile associated with increased Ras activity. This is the opposite observation for copy number events, as more events outside of *KRAS*, *NRAS*, and *HRAS* appear to confer lower scores, which may indicate either some sort of dosage response counteracting the effects of hyperactivation or alternative events that dampen accurate Ras classification. Furthermore, tumors harboring specific Ras pathway isoforms curated by the PanCanAtlas Pathways AWG are generally predicted to have higher scores than unconfirmed variants. We provide scores for all observed somatic Ras variants for TCGA tumors and CCLE cell lines at base-pair and amino-acid resolution (Data S4 and Data S5) and present this resource for potential follow-up study.

In conclusion, we presented a machine learning method to predict Ras activity in individual bulk tumors using transcriptomes. Our approach may sidestep requirements to profile multiple genomic measurements to detect Ras activation and identify more patients with activated Ras. Our approach can be used as an additional method to improve precision oncology (Cieślik and Chinnaiyan, 2018). Subclonal mutations may also prevent accurate Ras classification by gene sequencing. Training classifiers with single-cell RNA-seq data may enable the detection of rare events and can help to characterize intratumor heterogeneity. As data increase in scale and algorithms are better constructed to model disease heterogeneity, the ability to research downstream responses of pathway misregulation and identify multi-model therapies targeting various vulnerabilities of individual tumors will improve.

## STAR ★ METHODS

Detailed methods are provided in the online version of this paper and include the following:

### KEY RESOURCES TABLE

**Table:** 

REAGENT or RESOURCE	SOURCE	IDENTIFIER
Biological Samples		
KRAS mutant cell line profiles	NCBI Gene Expression Omnibus; Kim et al., 2017	GEO: GSE94937
Cancer Cell Line Encyclopedia Gene Expression	Barretina et al., 2012	CCLE
Cancer Cell Line Encyclopedia Mutations	Barretina et al., 2012	CCLE
Cancer Cell Line Encyclopedia Variants	Barretina et al., 2012	https://data.broadinstitute.org/ccle/CCLE_DepMap_18Q1_maf_20180207.txt
Deposited Data		
The Cancer Genome Atlas	Genome Data Commons	https://gdc.cancer.gov/about-data/publications/pancanatlas
Software and Algorithms		
Python v3.5.2	Python Core Team	https://www.python.org/
Sci-Kit Learn v0.18.1	Pedregosa et al., 2011	http://scikit-learn.org/
Pandas v0.20.3	McKinney 2010	http://pandas.pydata.org
Seaborn v0.7.1	Waskom et al., 2016 (https://doi.org/10.5281/zenodo.54844)	https://seaborn.pydata.org/
R v3.4.3	R Core Team	https://www.R-project.org
dplyr v0.7.1	Wickham et al., 2017	http://dplyr.tidyverse.org/
ggplot2 v2.2.1	Wickham 2009	http://ggplot2.tidyverse.org/
Custom Classifier Software	This paper	https://github.com/greenelab/pancancer
Other		
Curated Ras Pathway Genes	Sanchez-Vega et al., 2018	N/A
Curated Ras Pathway Variants	Chakravarty et al., 2017	http://oncokb.org/

### CONTACT FOR REAGENT AND RESOURCE SHARING

Further information and requests for resources and reagents should be directed to and will be fulfilled by the Lead Contact, Casey S. Greene (csgreene@upenn.edu). The Cancer Genome Atlas will provide instructions on how to access publicly available data.

### METHOD DETAILS

#### Training machine learning classifiers to detect aberrant gene events

We integrated Illumina RNaseq, multi-center mutation calls (MC3), and GISTIC2.0 copy number threshold calls from The Cancer Genome Atlas (TCGA) PanCanAtlas project to classify aberrant pathway function (Mermel et al., 2011). We downloaded TCGA data-sets from the Genome Data Commons (GDC). In total, there were 9,075 tumors that were measured on all three platforms that passed quality control filtering. We subset the gene expression matrix to the 8,000 most variably expressed genes by median absolute deviation (MAD), as genes that do not vary are unlikely to be useful for classification and to reduce training time. We dropped the target genes of interest (e.g., *KRAS*, *NRAS*, *HRAS* or *NF1*) when training the models to prevent the model from potentially relying too heavily on dosage-specific effects of these genes instead of the downstream response to their activation. We also removed the samples with the highest mutation burden to remove potential false positives. We defined these samples based on five standard deviations above the log10 total non-silent somatic mutation count per sample. Because we were interested in a balanced training set based on aberrant gene events, we further filtered samples to include only cancer-types with greater than 15 target gene events and a proportion of negatives to positives no less than 5%.

Using this data, we trained a supervised elastic net penalized logistic regression classifier with stochastic gradient descent (Zou and Hastie, 2005). Our model is trained on RNaseq gene expression (*X*) to predict gene status (*Y*) (see Figure 1). To control for tumors with a hypermutator phenotype and potential tissue-specific expression patterns, we included cancer-type dummy variables and per sample log10 mutation count in the model as covariates. We defined gold standard gene status using loss of function mutation and deep copy number losses for tumor suppressor genes and gain of function mutations and large copy number gains for oncogenes. For simplicity and to reduce the requirement for extensive manual curation, we considered any non-silent mutation including insertion-deletions in the gene body or mutations in splice site regions of target genes. For the specific focus of the paper, we integrated gain of function mutation and copy number gains for the oncogenes (*KRAS*, *NRAS*, and *HRAS*), and loss of function and deep copy number losses for the tumor suppressors (*NF1)*. For example, if a tumor had a deleterious mutation or copy number amplification in one of these genes, we considered the Ras status equal to one.

The objective of the classifier is to determine the probability a given sample (*i*) has a Ras event given the sample’s RNaseq measurements (*X_i_*). In order to achieve the objective, the classifier learns a vector of coefficients or gene-specific weights (*w*) that optimize the following penalized logistic function.

$$P(y_{i}=1|X_{i})=f(X_{i}w)=\frac{1}{1+e^{-wX_{i}}}\;\mathrm{negative}\;\mathrm{loglikelihood}=L=-\sum_{i=1}^{n}y_{i}\text{log}P(y_{i}=1|X_{i})+(1-y_{i})\text{log}P(y_{i}=0|X_{i})\;w=\mathrm{argmin}\left(L+\alpha \sum {|\left|w\right||}_{l}\right)$$

Where α and *l* are regularization and elastic net mixing hyperparameters that are only active during training, respectively. Using a training set consisting of 90% of the full dataset, equally balanced for different proportions of included cancer-types and Ras status, we performed cross validation over the hyperparameter grid: *l* = {0.15, 0.155, 0.16, 0.2, 0.25, 0.3, 0.4} and α = {0.1, 0.13, 0.15, 0.18, 0.2, 0.25, 0.3}. We used balanced 5-fold cross validation based on the highest cross-validation area under the receiver operating characteristic (AUROC).

We trained the Ras classifier using optimal hyperparameters (*l* = 0.15 and α = 0.1) and assessed performance on training, testing (held out 10% of data) and across 5-fold cross-validation intervals. In 5-fold cross-validation, the data are partitioned into five even sets (balanced by Ras status and cancer-type). Four of the folds, called training intervals, are used to construct the model. The model is then evaluated on the fifth fold, which is called the evaluation fold. The reported training performance comes from the folds used for training, while the cross-validation performance uses the evaluation fold. Therefore, performance on cross-validation intervals are the predictions reported on the training set samples when they were included in the internal cross-validation evaluation fold. The full model is reported in Data S3 and all resulting classification scores in Data S2 is the model learned from the training set alone.

#### Evaluating machine learning classifiers

We evaluated the pan-cancer classifiers in various ways. For every evaluation, we reported the AUROC and area under the precision-recall (AUPR) curve. We also compared gene specific classifiers built using pan-cancer data to classifiers trained independently using only data from individual cancer-types. In these cases, each cancer-type specific model was optimized individually. We compared how the pan-cancer model performed on individual cancer-types compared to individual cancer-type optimizations. Additionally, we cataloged the performance of the Ras classifier to predict *NF1* inactivation in various cancer-types. *NF1* is a tumor suppressor of Ras and we postulated that it would have similar downstream consequences that could be captured by the Ras classifier. Therefore, we performed the same procedure of filtering datasets and training pan and within cancer-type classifiers for *NF1*. We compared these *NF1* evaluations against the Ras classification. Lastly, we evaluated the Ras classifier on predicting aberrant mutations of other genes and variants in the Ras pathway and in two different cell line datasets.

#### Classifier Benchmarking Analyses

We determined the robustness of the classifier by evaluating performance under various input features and prediction tasks. We evaluated potential inflation of performance metrics by training a null model on a randomly shuffled input gene expression matrix. We did not shuffle the covariate information or the y matrix. Performance on the random shuffling of genes, while maintaining the same ratio of Ras mutations, provides insight into how the model would be expected to perform in a scenario lacking Ras activation signal. We also performed the same shuffling and classifier testing procedure as internal negative controls in every pan-cancer model and report ROC/PR curves and AUROC/AUPRs in each figure.

To assess value added in combining mutation and copy number data in the prediction task (altering the y matrix), we trained pan-cancer classifiers with the same procedure described above to predict Ras mutations and Ras copy number gains separately. The combined model presented here is the same model trained in Figure 2. To test the effect of dropping *KRAS*, *HRAS*, and *NRAS* from the model (altering the X matrix), we trained models with the previously described procedure with the input gene expression matrix without dropping Ras genes. We also tested a classifier after dropping 14 genes from the Expanded RASopathy Panel (Genetic Testing Registry). The genes included *BRAF*, *CBL*, *HRAS*, *KRAS*, *MAP2K1*, *MAP2K2*, *NF1*, *NRAS*, *PTPN11*, *RAF1*, *SHOC2*, *SOS1*, *SPRED1*, and *RIT1*. For the two previous comparisons, we compared the learned gene expression coefficients to the classifier trained in Figure 2. For the dropping genes analysis, we added back all dropped genes as zero weights. We also compared the performance of gene expression-only and covariate-only models (altering the X matrix) to the combined model presented in Figure 2. The y matrix remained the same, but each model was trained on only a subset of the combined X matrix. The differentially expressed genes visualized in Figure S2H were obtained from the differential expression analysis described below.

#### Differential Expression Analysis

We performed a differential expression analysis using the limma Bioconductor package (Ritchie et al., 2015). We adjusted the model by cancer-type by including cancer-type indicator variables in the limma design matrix. We considered all 9,074 samples and 20,500 genes in this analysis. We zero-one normalized the input matrix by gene prior to fitting with limma.

#### Cell Line Validation

We applied the Ras classifier to two independent cell line datasets. The first dataset was generated by Kim et al. (2017) and was deposited in the Gene Expression Omnibus (Edgar et al., 2002) with the identifier GEO: GSE94937. We used the preprocessed form of the data from (Kim et al., 2017). We also used data from 737 cell lines from the CCLE that had matching RNaseq and mutation data (Barretina et al., 2012). Of these 737, 708 also had variant level annotations. In order to apply the classifier to both cell-line data-sets, we z-score normalized gene expression values and subset the data to classifier genes, independently. 177 out of 185 (96%) of the features were in common to classifier genes in both datasets, so we proceeded to make predictions with this subset. In order to apply the predictions, we used the following transformation:

$$s=f(X_{i}w)=\frac{1}{1+e^{-wX}}$$

Where *s* is the classifier prediction, *w* is the gene weights, and *X* is the corresponding subset cell line gene expression matrix.

We used the CCLE pharmacologic profiling data, which measured the activity of 24 drugs across 504 CCLE cell lines (CCLE_NP24.2009_profiling_2012.02.20.csv). Data were accessed from https://portals.broadinstitute.org/ccle/data (Barretina et al., 2012).

#### Ras Pathway and Oncogenicity Curation

We used the PanCanAtlas Pathways Working Group definition of 38 core Ras pathway genes (Sanchez-Vega et al., 2018). We obtained oncogenicity assignments for mutations in these genes using OncoKB (Chakravarty et al., 2017) and additional manual curation by the PanCanAtlas Pathways AWG. The manual curation included referencing MutSig (Lawrence et al., 2013), hotspot analyses (Chang et al., 2016), and GISTIC Peaks (Mermel et al., 2011).

### QUANTIFICATION AND STATISTICAL ANALYSES

We performed all machine learning model training, testing, and evaluations using sci-kit learn (version 0.18.1) with python 3.5.2 (Pedregosa et al., 2011). We processed data using a combination of pandas (version 0.20.3) and dplyr (version 0.7.1) and visualized results using a combination of seaborn (version 0.7.1), ggplot2 (version 2.2.1), and PathwayMapper (Bahceci et al., 2017). R packages were run on R version 3.4.0. Please refer to the Key Resources Table and the available GitHub repository (https://github.com/greenelab/pancancer) for full software version details. We evaluated all classifiers using AUROC and AUPR. The AUROC is a metric describing the overall trade-off between true positive and false positive rates, while the AUPR measures precision against recall for a given classifier. An AUROC of 0.5 constitutes random guessing. We describe specific filtering steps for each analysis in various places in the Method Details section of the STAR Methods. We describe overall sample and gene filtering in the *Training* subsection. We discuss additional gene filtering for evaluating all alternative genes in the *Evaluation* subsection. We set random seeds in all computational analyses in order to preserve reproducibility. We performed independent t tests with unequal variances when comparing classifier scores for curated variants versus variants of unknown significance per Ras pathway gene. We performed the same test comparing CCLE cell line Ras classifier scores for Ras wild-type versus Ras (*KRAS*, *HRAS*, or *NRAS*) mutant samples and for Ras wild-type, *BRAF* wild-type versus Ras wild-type, *BRAF* mutant. Using the up to 388 cell lines with both gene expression and pharmacology data measured, we fit linear regression models comparing drug activity versus Ras classifier scores for all 24 drugs to Ras wild-type and Ras mutant cell lines individually. Using a Bonferroni adjusted p value (0.05/(24 * 2) = 0.001), we implicated two high correlated drugs (AZD6244 (Selumetinib) and PD-0325901). Selumetinib was tested on 387 cell lines while PD-0325901 was tested on 388 cell lines. We also used a chi square test for proportions of Ras mutations annotated as COSMIC variants in true positives compared to false negatives with a null hypothesis that both sets of samples have the same proportion of COSMIC variants.

### DATA AND SOFTWARE AVAILABILITY

All analytical results can be reproduced using the code available at https://github.com/greenelab/pancancer. There, we provide instructions to replicate the computing environment, download versioned data, and all scripts to reproduce the entire analysis pipeline. The pipeline is modular and amendable to generate classifiers and predictions for any combination of genes, pathways, and TCGA PanCanAtlas cancer-types. The source code has been deposited to Zenodo at https://doi.org/10.5281/zenodo.1186801.

## Supplementary Material

1

2

3

4

5

6

7

## Highlights

- Machine learning models of gene expression can aid in precision oncology
- The model can identify Ras activation in TCGA PanCanAtlas tumors and cell lines
- The model identifies events that phenocopy Ras activation, such as NF1 loss
- The approach characterizes Ras pathway variants and is extensible to other pathways
