# Supplementary material for: Machine Learning Detects Pan-cancer Ras Pathway Activation in The Cancer Genome Atlas
Source: Cell Rep. Author manuscript; Available in PMC 2018 Apr 26. (PMC5918694; doi:10.1016/j.celrep.2018.03.046)
Supplement: 1 [file NIHMS958974-supplement-1.pdf]

Cell Reports, Volume *23*

Supplemental Information

Machine Learning Detects

Pan-cancer Ras Pathway Activation

in The Cancer Genome Atlas

Gregory P. Way, Francisco Sanchez-Vega, Konnor La, Joshua Armenia, Walid K. Chatila, Augustin Luna, Chris Sander, Andrew D. Cherniack, Marco Mina, Giovanni Ciriello, Nikolaus Schultz, The Cancer Genome Atlas Research Network, Yolanda Sanchez, and Casey S. Greene

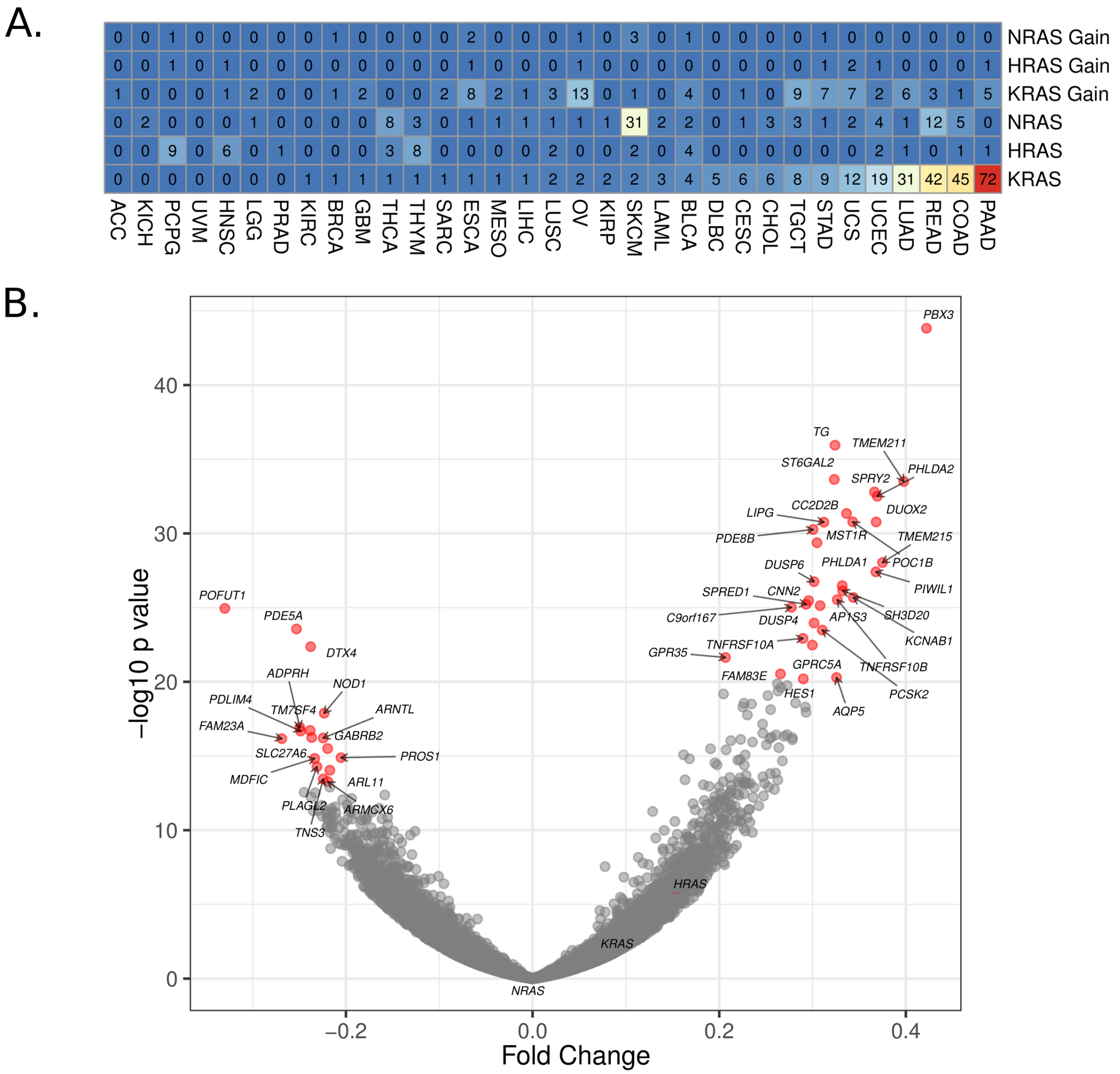

Figure S1. *Ras pathway alteration percentages in TCGA PanCanAtlas; related to Figure 2 and Data S1.* **(A)** Percentage of *KRAS*, *HRAS*, and *NRAS* mutations and copy number gains across 33 different cancer-types from TCGA PanCanAtlas. **(B)** Differentially expressed genes between Ras aberrant and Ras wild-type PanCanAtlas tumors. Analysis is controlled for cancer-type.

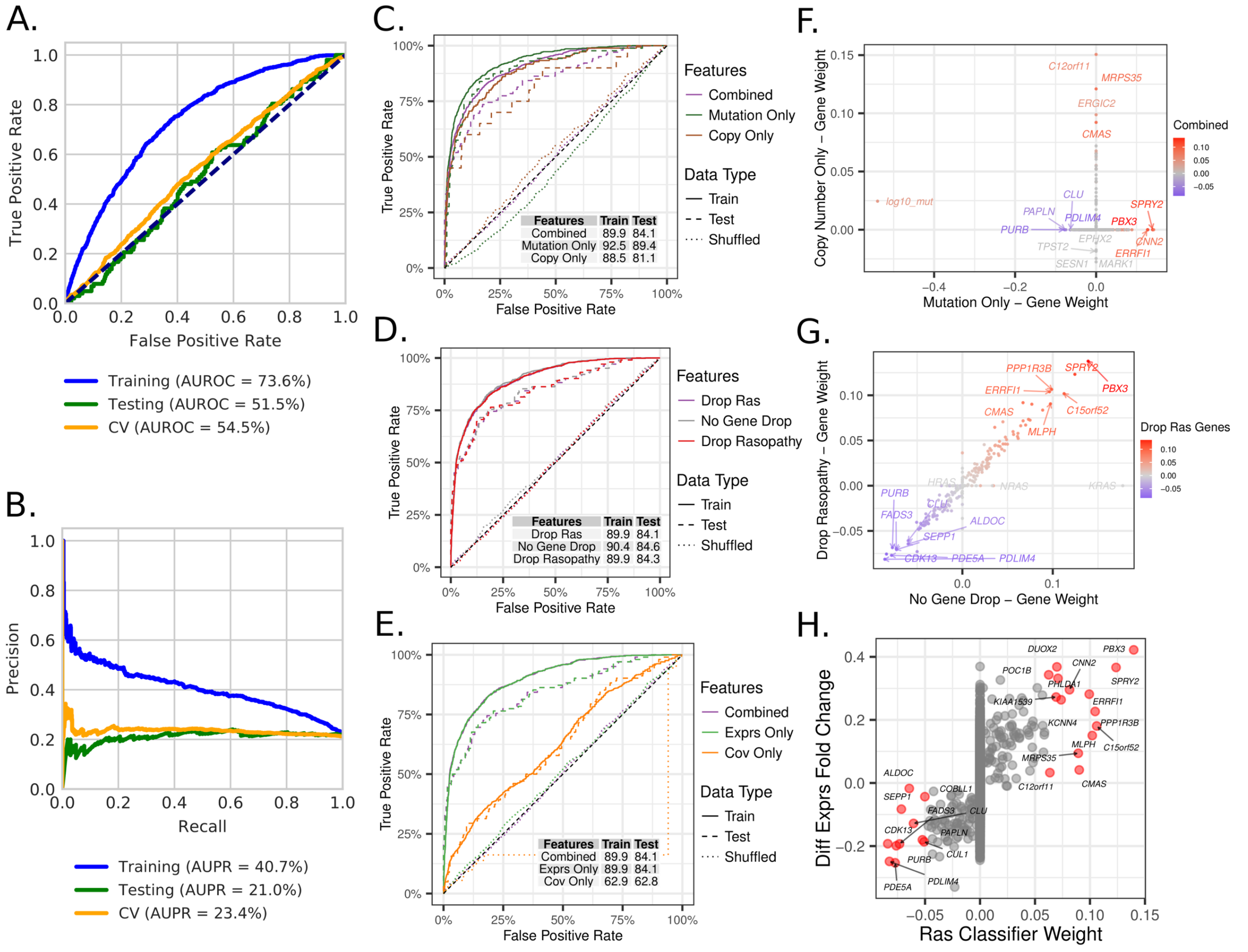

Figure S2. *Benchmarking PanCancer Ras Classifiers; related to Figure 2.* **(A)** Receiver operating characteristic (ROC) curve and **(B)** Precision recall (PR) curve for a null model trained on a randomly shuffled RNAseq matrix. Also provided are the area under the ROC (AUROC) and area under the PR (AUPR) curves for training, testing, and cross validation sets. **(C)** ROC curve for three models predicting: 1) Ras mutations only; 2) Ras copy number gains only; 3) Combined data (model in Figure 2). The AUROC is provided for both training and testing sets. **(D)** ROC/AUROC across train and test sets for dropping different genes from the RNAseq matrix. The Drop Ras model is the model provided in Figure 2. **(E)** ROC/AUROC across train and test sets for using expression data or covariates only. The combined model is the model provided in Figure 2. In all ROC curves, the dashed navy line represents a hypothetical random guess classifier. Gene coefficients for the models presented in **(F)** panel C and in **(G)** panel D. The points are colored by the model presented in Figure 2. **(H)** Differential fold change for tumors with active Ras against tumors with wild-type Ras compared against the Ras classifier gene coefficients provided in Figure 2. Red points correspond to labelled genes.

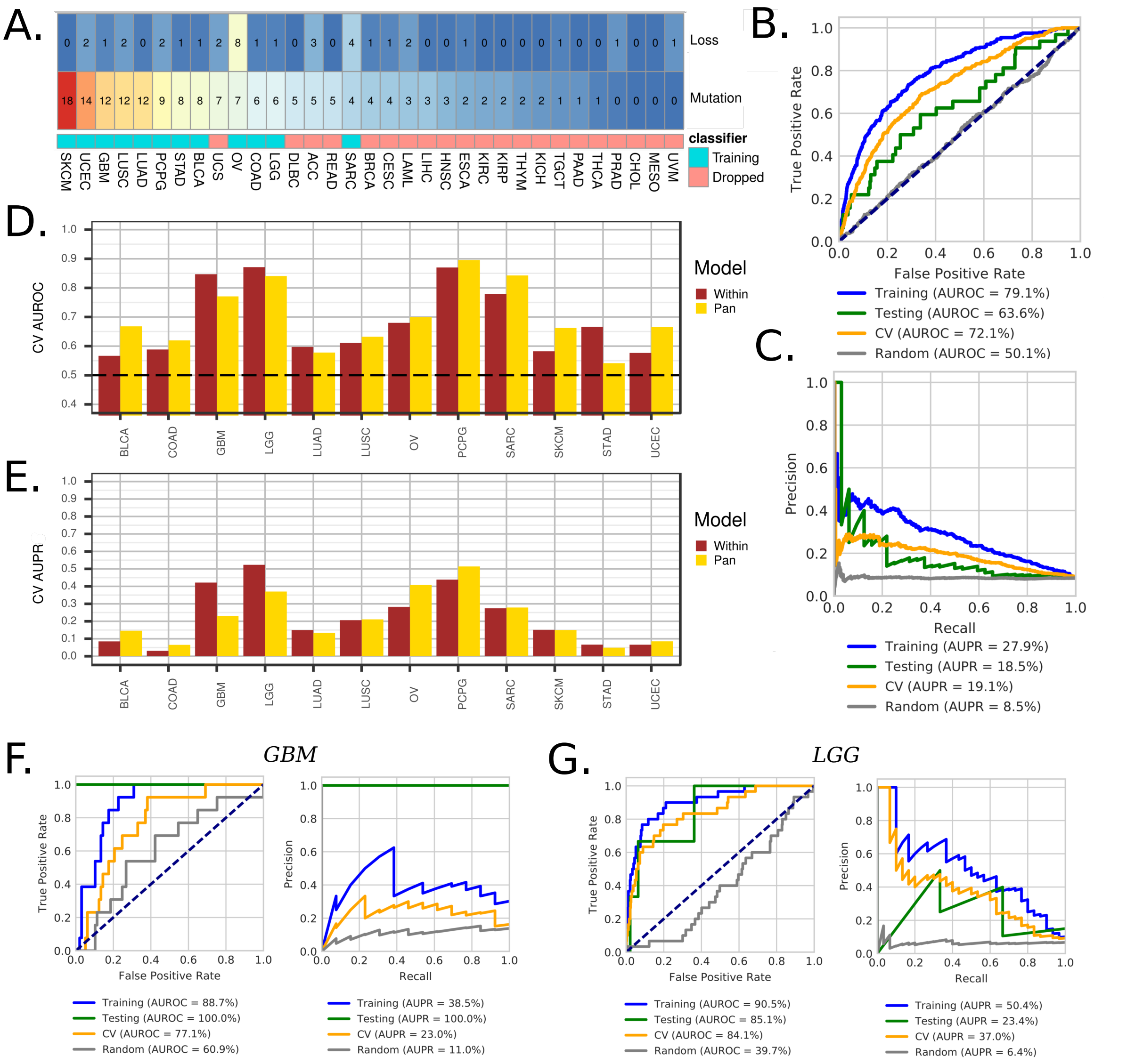

Figure S3. *Pan-cancer NF1 classification performance; related to Figures 2 and 4A.* **(A)** Cancer-type specific percentages of *NF1* inactivation by copy number loss and deleterious mutation. The colored squares indicate if the cancer type was included in model training. **(B)** Receiver operating characteristic (ROC) curve and Area under the ROC curve (AUROC) given for training, testing, and cross-validation (CV) sets. **(C)** Precision Recall (PR) Curve and corresponding area under the PR (AUPR) curve for each evaluation set. Cancer-type specific CV **(D)** AUROC and **(E)** AUPR for the *NF1* pan-cancer model compared to separate models trained on each cancer type independently. ROC and PR curves for predicting *NF1* inactivation in **(F)** GBM and **(G)** LGG using the pan-cancer model. The grey lines represent predictions made on a shuffled gene expression matrix.

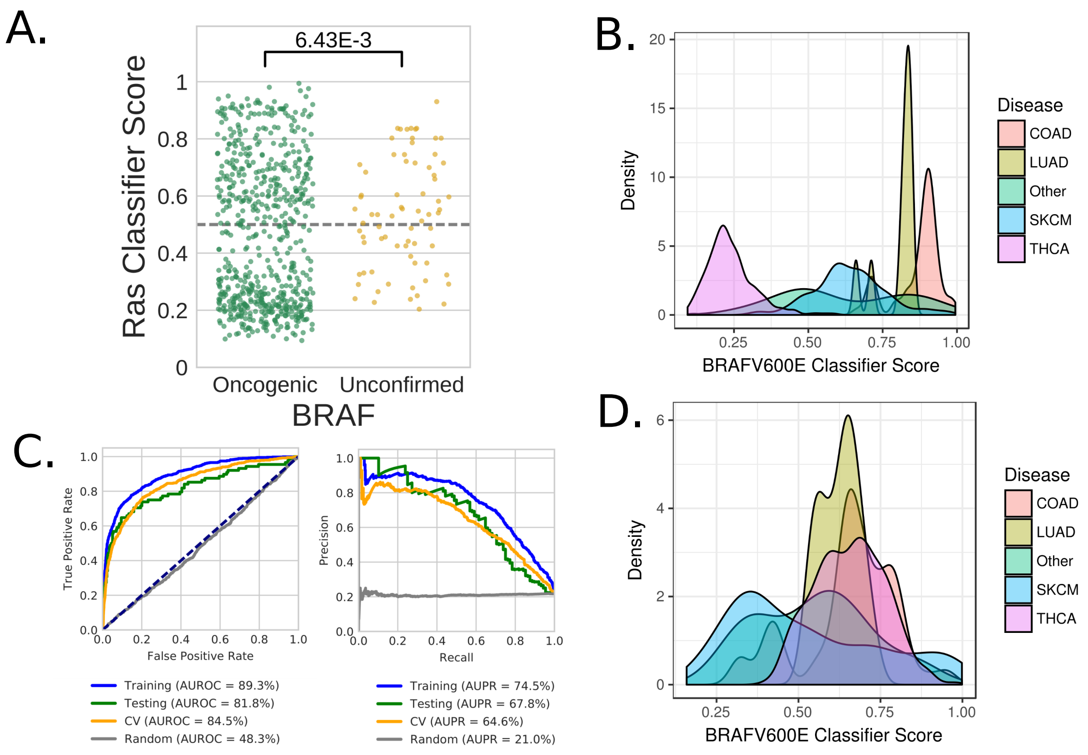

Figure S4. *Predicting BRAF with the Ras Classifier; related to Figure 2.* **(A)** Predictions for tumors with oncogenic or unconfirmed variants in *BRAF* given by the Ras classifier evaluated in Figure 2. **(B)** Ras classifier scores assigned to samples with *BRAF* V600E mutations stratified by cancer type. A score above 0.5 indicates a prediction of activated Ras. **(C)** Ras classifier evaluation after removing THCA and SKCM from training. ROC and PR curves for the Ras classifier without THCA and SKCM does not indicate reduced performance. The grey lines represent predictions made on a shuffled gene expression matrix. **(D)** Ras classifier without THCA and SKCM classify *BRAF* V600E as Ras wildtype in THCA, but not in SKCM.
